# Reperfusion Strategies in Donation After Circulatory Death Heart Transplantation: An Up-to-Date Narrative Review

**DOI:** 10.3390/jcm15155947

**Published:** 2026-07-30

**Authors:** Antonella Galeone, Giovanni Battista Luciani, Francesco Onorati

**Affiliations:** 1 Department of Surgery, Dentistry, Pediatrics and Gynecology, Division of Cardiac Surgery, University of Verona, 37126 Verona, Italy; 2 Cardiac Surgery Unit, Ospedale Santa Chiara, University of Trento, 38122 Trento, Italy

**Keywords:** donation after circulatory death, heart transplantation, direct procurement, normothermic regional perfusion, machine perfusion

## Abstract

Donation after circulatory death has emerged worldwide as a safe and effective alternative pathway to increase the donor pool. However, organs undergo a variable period of warm ischemia during the agonal phase and the cardiac arrest before graft procurement, and the heart is particularly vulnerable to injury provoked by warm ischemia and subsequent reperfusion. Several reperfusion strategies are currently available for the recovery and assessment of heart function in these donors, with promising early and long-term results. Nevertheless, each technique implies specific financial, logistical and ethical challenges, translating into a heterogeneous landscape of protocols across European and non-European countries. Additionally, no randomized clinical trials have been published to assess the superiority of one technique over the other, and current clinical evidence is based on retrospective registry data and single-center observational studies.

## 1. Introduction

**Figure 1:**
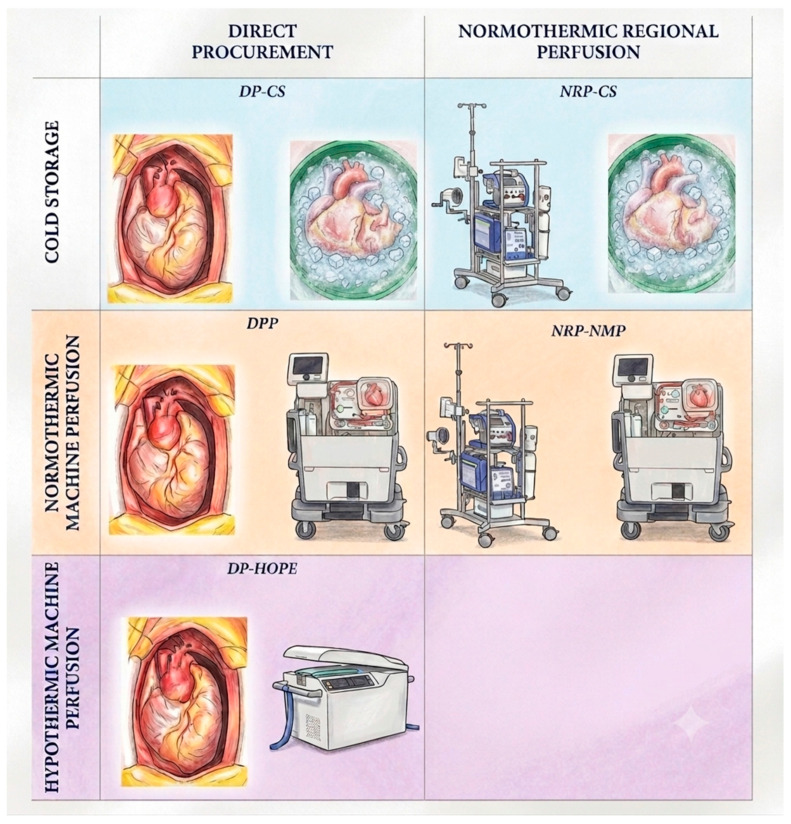
Schematic illustration of the various reperfusion strategies in cDCD heart transplantation. CS: cold storage; DP: direct procurement, DPP: direct procurement and perfusion; HOPE: Hypothermic oxygenated perfusion; NMP: normothermic machine perfusion; NRP: normothermic regional perfusion.

Heart transplantation (HT) represents the gold standard for the treatment of end-stage heart failure, providing better survival rates and quality of life scores [1]. However, not all patients may benefit from HT because of the discrepancy between the growing number of potential heart recipients due to an aging population and better heart failure care [2] and the organ donor shortage. This discrepancy implies an overall mortality rate of 7% while on a waiting list that has significantly improved in the recent era but is not negligible yet [3]. One strategy to address the limited organ availability is to expand criteria for the acceptance of organ donors [4,5,6]. Donation after circulatory death (DCD), in addition to donation after brain death (DBD), also represents a promising possibility to increase the donor pool. DCD transplant programs are increasing worldwide [7] and currently DCD donation accounts for more than 50% of all deceased donors in many European countries, such as Belgium, the Netherlands and the United Kingdom (UK) [8,9]. Previous studies showed that the adoption of a DCD heart transplant program could increase adult heart transplantation volume by approximately 15–30% [10], reduce the mortality on the waiting list by 40% [11] and reduce the waitlist time [12]. However, unlike conventional DBD, in which donor organs are continuously perfused until aortic cross-clamping and infusion of preservation solutions, DCD organs undergo a variable period of warm ischemia during the agonal phase and the cardiac arrest before graft procurement. The heart is particularly vulnerable to injury provoked by warm ischemia and subsequent reperfusion [13], raising concerns about DCD graft functionality and quality [14]. An experimental study based on endomyocardial biopsies of non-cardiac DCD donors aimed to identify the time point when myocardial function decreases and the risk of graft dysfunction increases through in vivo close monitoring of biochemical changes in cardiac myocytes during the process of DCD [15]. The authors showed a reduction in contractility and mitochondrial activity and an increase in apoptosis beyond 10 min of cardiac arrest, indicating a significant compromise in cellular function and viability [15]. Many techniques have been proposed to reperfuse, recover and assess cardiac function after the warm ischemic period (Figure 1). The aim of this review is to describe all available reperfusion strategies currently used for the recovery and assessment of cardiac function in DCD donors, including the pros and cons of each technique, as well as early and long-term results associated with these techniques.

## 2. Historical Background

Human organ transplantation developed in the 1960s using organs from donors declared dead using circulatory criteria [16]. Historically, the world’s first human-to-human orthotopic heart transplantation was performed by Christian Barnard in December 1967 using a donor declared dead with circulatory criteria [17]. A young white woman sustained a massive head injury after being hit by a car and was certified as having a lethal brain injury without any chance of recovery by the neurosurgeon who had been called to treat the patient and who eventually referred her as an organ donor. As there were no laws relating to brain death and organ transplantation in South Africa or elsewhere, Barnard invited the State’s forensic pathologist to the operating theatre (OT). The donor was prepared and draped for surgery, ventilation was discontinued, and their blood pressure dropped, followed by cardiac arrest. After 5 min of no activity on the electrocardiogram (ECG) and the ascertainment of the absence of any spontaneous respiratory movements and reflexes, the medical examiner pronounced that death had occurred. Barnard’s assistants then rapidly opened the chest, initiated cardiopulmonary bypass, cooled the heart to a low temperature and proceeded with heart procurement. The recipient had been prepared in the adjacent OT and Barnard proceeded with the transplant. In August 1968, an ad hoc committee at Harvard Medical School published a landmark document suggesting that death should be declared in case of irreversible cessation of the function of the entire brain, including the brainstem [18]. In addition to the traditional way of defining death as irreversible cessation of circulatory and respiratory functions, the committee proposed a new definition of death (i.e., brain death) that focused on the loss of neurological function. This report was codified into United States (US) law in 1981, as the Uniform Determination of Death Act, which established brain death as legal death, and most European countries subsequently accepted it. Following the first publication of the definition of brain death and the criteria to ascertain brain death by the Harvard Medical School, most organ transplants have been performed with organs from donors declared dead using neurological criteria.

## 3. DCD Donors and DCD Process

DCD donors can be classified into four categories according to the Maastricht classification of 1994 [19], subsequently modified at the 6th International Conference on Organ Donation after Circulatory Death held in Paris in 2013 [20]. Countries such as Belgium, the Netherlands, Spain and Canada, which have a legal framework for medically assisted death, may benefit from a fifth category for donation after euthanasia [21]. DCD can also be considered, according to conditions surrounding circulatory arrest, as uncontrolled or controlled. Controlled DCD (cDCD) is of particular interest for heart transplantation because the precise course of circulatory arrest can be followed and, consequently, the duration and conditions of warm ischemia are known (Table 1).

**Table 1:** Categories of DCD donors according to modified Maastricht classification.

Category	Circumstances	Cardiac Arrest	Type
**I**	Found dead/dead on arrival	Unwitnessed	Uncontrolled
**II**	Unsuccessful resuscitation	Witnessed	Uncontrolled
**III**	Withdrawal of life-sustaining therapy	Awaited	Controlled
**IV**	After brain death declaration	Unexpected	Controlled/uncontrolled
**V**	Euthanasia	Awaited	Controlled

**Figure 2:**
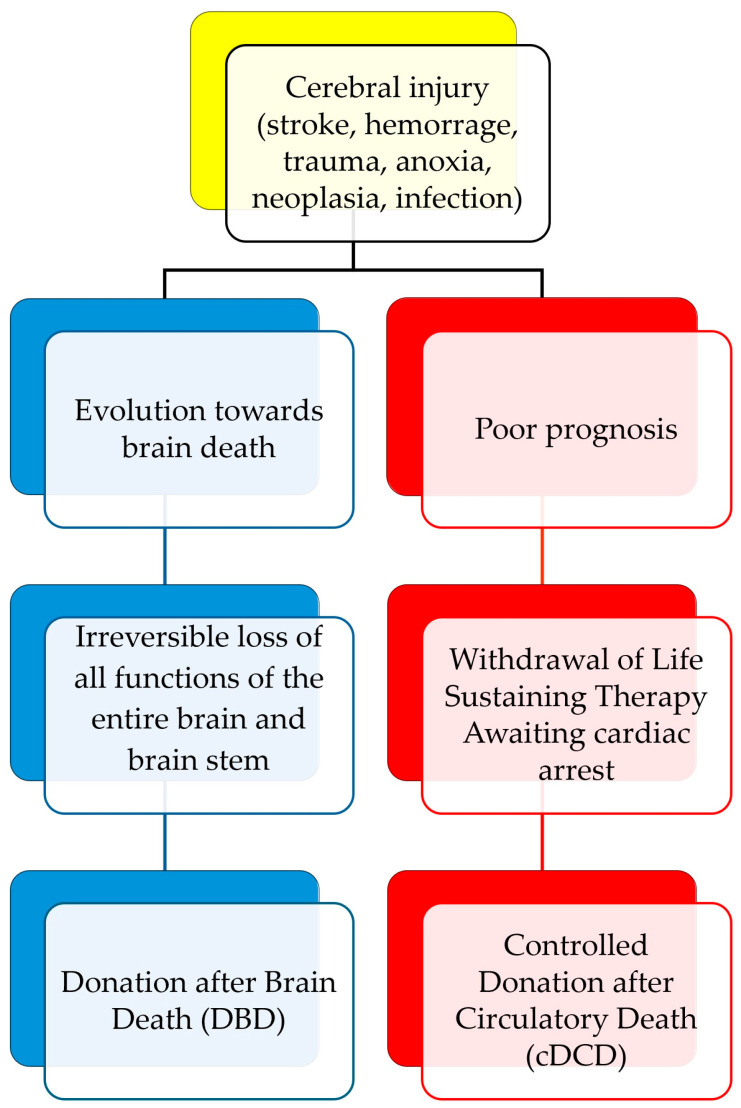
Donation after brain death (DBD) and controlled donation after circulatory death (cDCD).

The third category refers to patients with catastrophic cerebral lesions and poor prognosis that do not fulfil all the neurological criteria required for brain death diagnosis. In these patients, withdrawal of life-sustaining therapy (WLST) is planned (Figure 2). Afterwards, according to the patient’s and/or family’s wish to donate, a proposal for organ donation could be discussed.

**Figure 3:**
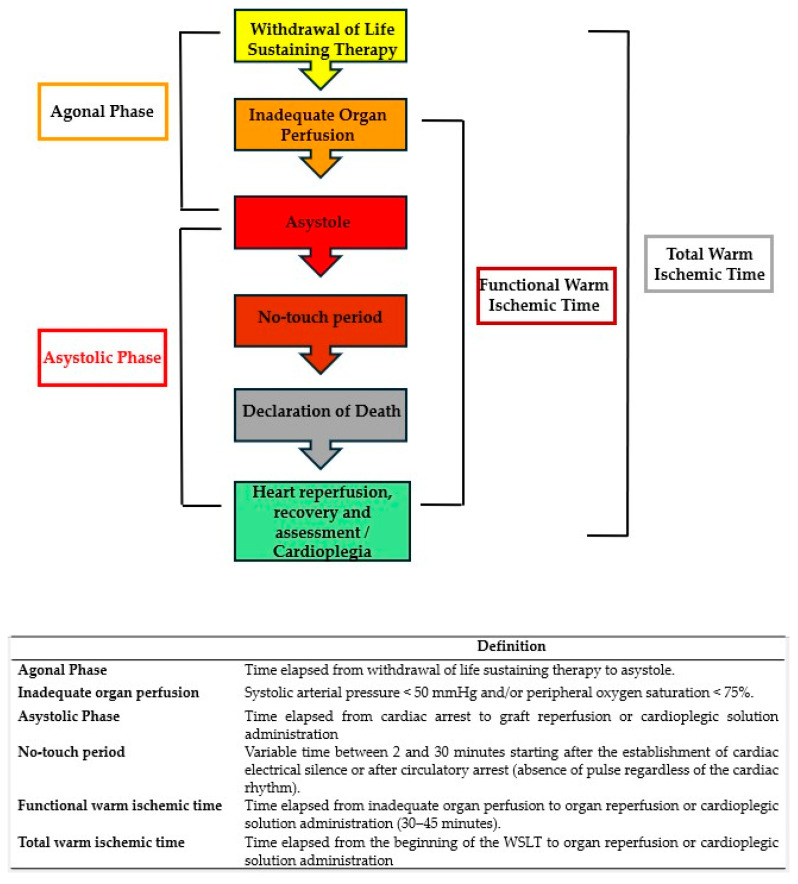
Flowchart of the DCD process.

WSLT is usually followed by cardiac arrest. In Maastricht III donors, DCD is controlled as the cardiac arrest is awaited. Time elapsed from WSLT to asystole is called the agonal phase and may have variable duration. If cardiac arrest does not occur within a defined length of time following WLST, typically sixty minutes, the donation process is stopped, because during this phase thoracoabdominal organs are subjected to a warm ischemic time due to inadequate perfusion, which may damage organs and compromise their quality. Conventionally, inadequate organ perfusion is defined as systolic arterial pressure < 50 mmHg and/or peripheral oxygen saturation < 75%. In most countries, heparin is administered *ante mortem* when inadequate perfusion occurs. After cardiac arrest, it is necessary to observe a no-touch period that varies from 2 to 30 min across European countries [7] to ensure that autoresuscitation will not occur. Previous research showed that autoresuscitation between 2 and 5 min is rare [22]. Although there are some international variations in the circulatory determination of death [7], there is general consensus that absence of pulse is a necessary criterion and that the circulatory determination of death requires absence of circulation for 5 min. It is not necessary, or desirable, to extend the period of observation beyond 5 min of circulatory arrest. Besides, electrical asystole is not required for the declaration of death in many countries, as electrical activity may continue for several minutes after cessation of circulation. In Italy, on the contrary, the declaration of death with cardiac criteria requires continuous ECG recording showing the absence of any cardiac electrical activity for at least twenty minutes. In addition, the no-touch period must start after the establishment of cardiac electrical silence, rather than after circulatory arrest (absence of pulse regardless of the cardiac rhythm) [23]. Both these requirements may significantly prolong the ischemic time during the cDCD process and further damage organs. The asystolic phase is defined as the time elapsed from cardiac arrest to graft reperfusion or cardioplegic solution administration, based on the technique used. Functional warm ischemic time (fWIT) is defined as the time elapsed from inadequate organ perfusion to organ reperfusion or cardioplegic solution administration, and should be less than 30 min. In countries that require a longer no-touch period, longer fWIT up to 45 min is tolerated [23]. Total WIT (tWIT) is defined as the time elapsed from the beginning of the WSLT to organ reperfusion or cardioplegic solution administration. After the declaration of death, it is possible to proceed with surgical techniques aimed at reperfusion, recovery and assessment of the heart, which will be discussed below. The cDCD process is illustrated in Figure 3.

## 4. Direct Procurement and Cold Storage

After the initial experience in South Africa, DCD donors were abandoned for heart transplantation until 2004, when Boucek reintroduced the use of DCD donors and published, in 2008, the results of three pediatric heart transplants performed in Denver between 2004 and 2007, using the technique of *direct procurement and cold storage* (DP-CS) in co-located neonatal cDCD donors [24]. The neonates underwent WLST in the OT, after the placement of femoral venous and arterial sheaths. The extubation was followed by heparin administration (300 UI/kg). When cardiocirculatory function ceased, the observation period before death declaration was 3 min for the first patient and 75 s for the other two patients, according to the recommendations of the local ethics committee. If death occurred within 30 min after extubation, the patient was considered suitable for donation, and cold preservation fluid (30–50 mL/kg) was infused through the distal port of a balloon arterial catheter placed in the ascending aorta. A median sternotomy was simultaneously performed, topical cooling was begun and the inferior vena cava was rapidly opened to prevent cardiac distention.

The DP-CS requires the co-localization of donor and recipient to reduce the ischemic time. One of the main concerns with the DP-CS technique is that it does not allow the evaluation of the graft function prior to implantation in the recipient. In the series reported by Boucek, however, all three pediatric heart transplant recipients survived to 6 months [24].

## 5. Direct Procurement and Perfusion

In 2015, Dhital and colleagues published the results of the Australian experience with adult heart transplantation using cDCD donors distantly procured using the technique of *direct procurement and perfusion* (DPP) [25]. This technique consists of direct procurement and *ex situ* normothermic perfusion (NMP) with the Organ Care System (OCS^TM^, TransMedics^®^, Inc., Andover, MA, USA) for the recovery and assessment of the heart [26]. In their initial experience on three adult donors, the authors considered Maastricht category III donors aged younger than 40 years with less than 30 min from WLST to delivery of cardioplegia. The donor age limit of 40 years was chosen to minimise the risk of procuring hearts with a pre-existing disease and because of concerns about the tolerability of ischemic injury in hearts from older donors [27], and the 30 min warm ischaemic time was chosen based on preclinical studies [28]. Subsequently, the donor age upper limit was raised to 55 years. The WSLT was done in the intensive care unit. The observation period after cessation of circulation varies in Australia and is at least 2 min in New South Wales and 5 min in other states. At the end of the observation period, the donors were declared deceased and quickly transferred to the OT, where they were intubated and prepared for surgery. A median sternotomy and laparotomy were done simultaneously, and a large venous cannula was placed into the right atrium to rapidly collect 1.5 L of blood to prime the *ex situ* perfusion device. Heparin was only added to the blood collection bag and not administered to the donor, according to New South Wales state regulations on the DCD process. The OCS circuit prime contained 1.5 L of donor blood that had been passed through a leucocyte filter and 500 mL of Transmedics^®^ priming solution including buffered electrolytes, mannitol, vitamins, and steroids. After aortic cross-clamping, 1 L of cold crystalloid St Thomas’ cardioplegia was delivered in the aortic root. Left atrial appendage and the inferior vena cava were cut to ensure heart venting, and the donor cardiectomy was performed in a standard fashion. The aorta and the pulmonary artery were then cannulated, and the donor heart was attached to the OCS. The heart was positioned in the NMP so that oxygenated blood directly entered the ascending aorta in a retrograde manner and then the coronary arteries. Blood then returned to the right side of the heart and was diverted through a cannula placed in the pulmonary artery before draining into the circuit reservoir. A vent was placed in the left atrium to decompress the left ventricle, and then the superior vena cava and inferior vena cava were both closed. Electrical cardioversion can be used in case of ventricular fibrillation after reperfusion, and biventricular epicardial pacing wires can be used in case of bradycardia. Aortic pressure, coronary flow, and arterio-venous lactate concentrations are used to assess cardiac function, with a lower venous concentration indicating lactate uptake and therefore satisfactory myocardial function. A Transmedics^®^ maintenance solution containing isotonic electrolytes, amino acids, dextrose–insulin, and low-dose adenosine was infused at a rate of 0–30 mL/h during *ex situ* perfusion to keep coronary flow within a range of 650–900 mL/min. Another infusion containing adrenaline was used to control coronary vascular resistance and heart rate to keep aortic pressure between 65 and 90 mm Hg and heart rate between 65 and 100 beats per min. A total concentration of lactate of less than 5 mmol/L in the perfusate combined with myocardial lactate extraction (coronary inflow lactate > coronary effluent lactate) was considered evidence of myocardial viability [29]. The results of the initial 23 heart transplants performed in Australia using cDCD donors with the DPP technique showed excellent 4-year survival outcomes; however, in this series, extracorporeal membrane oxygenation (ECMO) for severe primary graft dysfunction (PGD) was required in eight (35%) recipients [30]. The review of the first 8 years of heart transplant activity using cDCD donors and DPP technique in 74 recipients demonstrated a significant reduction in the incidence of severe PGD from 35% to 8%. Severe PGD was associated only with the asystolic warm ischemic time [31]. Heart transplantation from cDCD donors allowed an increase in overall heart transplant activity by 25%, with similar outcomes compared to DBD donors. In fact, 1- and 5-year survival of cDCD heart transplant recipients were 94% and 88%, respectively, and were comparable to that of a contemporary cohort of DBD recipients [31]. Recently, the 10-year experience with the DPP technique has been reported. A long-term outcomes analysis of 118 cDCD and 385 DBD heart transplants performed from July 2014 to December 2024 showed that recipients from cDCD donors have similar survival and freedom from CAV at 10 years compared with recipients from DBD donors [32].

In Europe, the DPP technique has been adopted in Austria and in the Netherlands [33]. In the US, the first adult cDCD heart transplant was performed in 2019 as part of a randomized controlled trial (RCT) comparing cDCD heart transplants using DPP to DBD using CS [34]. The trial included Maastricht category III donors, aged 18–49 years, with a WIT ≤ 30 min. A total of 180 patients were randomized (90 in the DCD-OCS group and 90 in the DBD-CS control group) and transplanted at 13 heart transplant centers in the US between 2019 and 2020. A total of 101 cDCD donor hearts were instrumented on OCS; of those, 90 were transplanted, with an overall cDCD heart utilization rate of 89%. One-year patient survival was 93.3% in the DCD group and 87.3% in the DBD group. Incidence of moderate or severe ISHLT PGD was 20% in the DCD group and 9.1% in the DBD group. Thus, the trial demonstrated that the use of OCS resulted in a high rate of DCD heart utilization for transplantation with excellent patient and graft survival outcomes compared to DBD donor hearts [35].

## 6. Direct Procurement and Hypothermic Oxygenated Perfusion

A new strategy using *direct procurement and hypothermic oxygenated perfusion* (DP-HOPE) with the XVIVO Heart Preservation System (XVIVO, Mölndal, Sweden) has recently been proposed as an effective alternative for cDCD heart transplantation [36]. The XVIVO system is a disposable perfusion unit that continuously perfuses the heart with a cold (8 °C) oxygenated cardioplegic solution supplemented with red blood cells (RBCs). The XVIVO system consists of a reservoir, a pressure-controlled roller pump, an oxygenator, an arterial-leukocyte filter, a heater–cooler unit, oxygen and carbon dioxide containers, a gas mixer, sensors, and a programmable control system. The system is primed with 2.5 L of an albumin-based hyperoncotic, cardioplegic perfusion solution and 300 to 500 mL of type O or recipient-matched blood to provide a 10% to 15% hematocrit [37]. After aortic cross-clamping, the heart is arrested by the heart preservation solution without RBCs, and after standard cardiectomy, the ascending aorta is cannulated with a self-deairing aortic cannula secured with a band tie. A silicon tube is placed through the mitral valve into the left ventricle and sutured to the left atrial wall to prevent left ventricular distension during perfusion. The heart is placed in the reservoir and submerged into the preservation solution. Following the deairing procedure, the heart is perfused through the aortic cannula to the coronary arteries at a mean aortic root pressure of 20 mm H, providing a coronary flow between 150 and 250 mL/min, at a temperature of 8 °C in a non-beating static state, which also reduces metabolic heart requirements. However, this technique does not allow formal functional assessment after warm ischemia.

### 6.1. HOPE in DBD Heart Transplantation

Preliminary results on the feasibility and safety of the XVIVO device for clinical use in heart transplantation were provided by a nonrandomized phase II study in which 25 DBD donors were assigned to static cold storage (SCS) and 6 to XVIVO non-ischemic hypothermic perfusion [37]. The authors found no deaths or cardiac-related adverse events in the XVIVO group, and four deaths within six months after transplantation and three cardiac-related adverse events in the SCS group. A multicenter, randomized, controlled, open-label clinical trial with a superiority design enrolled 229 patients in 15 centers across eight European countries who underwent heart transplantation with a DBD donor and were randomly assigned to SCS or HOPE [38]. The primary outcome was time to first event of a composite of either cardiac-related death, moderate or severe PGD of the left ventricle, PGD of the right ventricle, acute cellular rejection at least grade 2R, or graft failure (with use of mechanical circulatory support or re-transplantation) within 30 days after transplantation. The primary analysis population included 204 patients who received a heart transplant. The primary endpoint was registered in 19 (19%) of 101 patients in the HOPE group and 31 (30%) of 103 patients in the SCS group, corresponding to a risk reduction of 44%. PGD was the primary outcome event in 11 (11%) patients in the HOPE group and 29 (28%) in the SCS group [38]. Of note, this technology has also been used for organ preservation in the first-in-human heart xenotransplantation [39]. Further reports showed that HOPE provides safe and effective preservation of the graft for very long periods, allowing retrieval from remote geographic locations. A non-randomized, single-arm, multicenter investigation was conducted in Australia and New Zealand to verify the effect of HOPE on DBD donor hearts with a preservation time of 6 to 8 h, showing 100% recipient survival at 30 days and PGD in only one long-preservation-time recipient [40]. A case report reported the first instance of a DBD donor heart being flown across the Atlantic on a commercial airline flight, covering 6750 km from the French West Indies to Paris, which did not develop any graft dysfunction after 12 h of preservation with the XVIVO system [41].

### 6.2. DP-HOPE in cDCD Heart Transplantation

Finally, the DP-HOPE technique has been applied to humans for DCD heart transplantation. In a series of three cases, the authors reported 100% recipient survival at 30 days and no PGD or need for mechanical circulatory support [36]. Noteworthy, in this series FWIT was <30 min and donors’ age was <55 years, and the longest ischemic time was 6 h. Recently, the first North American experience using DP-HOPE for the recovery of DCD cardiac allografts has been published [42]. In this series, five patients underwent orthotopic heart transplantation using DCD allografts preserved with HOPE, with an average preservation time of 298 min. One patient died because of multiple extracardiac complications, but with preserved allograft function, no case of severe PGD was observed, and one patient required mechanical circulatory support for secondary graft dysfunction. The world’s first experience with the HOPE technique in pediatric heart transplant has been recently reported. Hearts recovered from six pediatric donors (three DCD and three DBD), aged 16 months–13 years. In the DCD cohort, median fWIT was 19 min, and there was no severe PGD. Survival at a median follow-up of 287.5 days was 100%, and all grafts had normal systolic function at echocardiography [43]. It must be noted that the use of DP-HOPE in DCD heart transplants does not allow any formal evaluation of the heart after it was exposed to the obligatory period of warm ischemia; however, results in adult and pediatric recipients are very promising, challenging NMP’s established role as the default strategy for higher-risk organs [44]. Of note, the XVIVO heart technology is not yet commercially available, and it is currently an investigational device strictly limited to ongoing clinical trials and compassionate use.

## 7. Normothermic Regional Perfusion and Machine Perfusion or Cold Storage

In 2015, the Royal Papworth Hospital performed the first cDCD adult heart transplant in Europe using *in situ* resuscitation of the donor heart with *normothermic regional perfusion* followed by *ex situ machine perfusion (NRP-MP)* [45]. The protocol included Maastricht III donors aged 18–57 years, with an expected death within 4 h from WSLT. Following the mechanical asystole, the 5 min no-touch period and the declaration of death, the donor was transferred to the OT, where a median sternotomy was performed, 30,000 UI of heparin was injected into the heart, and the supra-aortic trunks were cross-clamped to prevent cerebral perfusion. The ascending aorta and the right atrium were cannulated and connected to a circuit consisting of a centrifugal pump and an oxygenator to establish thoraco-abdominal NRP (TA-NRP) with a target flow rate of 5 L/min, a mean arterial pressure > 50 mmHg and a temperature > 35 °C. Once cardiac contractility had recovered, the donor was weaned off NRP. A pulmonary artery catheter was inserted to measure cardiac output and pulmonary pressures, and transesophageal echocardiography was performed to assess biventricular and valve function. The graft was considered suitable for HT if the following criteria were met: cardiac index (CI) > 2.5 L/min/m2, central venous pressure (CVP) < 12 mmHg, pulmonary capillary wedge pressure (PCWP) < 12 mmHg, left ventricular ejection fraction (LVEF) > 50% [45]. The donor heart was procured with crystalloid cardioplegic solution and transported using machine perfusion with the OCS. Later, the same authors published their experience with NRP followed by graft preservation in cold storage in the case of co-localization of the donor and the recipient (NRP-CS) [46]. The results of the first 26 cDCD heart transplants (12 NRP and 14 DPP) performed at the Royal Papworth Hospital from 2015 to 2017 showed that 90-day survival was not significantly different between cDCD and matched DBD transplant recipients (92% vs. 96%, respectively) [47]. The introduction of a cDCD heart transplant program increased heart transplant activity by 48% in 5 years, with no difference in 30-day and 1-year survival between 79 cDCD (19 NRP-MP, 3 NRP-CS, 55 DPP) and 79 DBD matched recipients [48].

More recent experiences in other European countries have shown that the use of TA-NRP followed by CS, without the need for an *ex situ* heart perfusion device, reduces the complexity of the procedure and the overall costs involved, without compromising the outcomes of the HT. The NRP-CS technique has been used in Belgium since 2019 with some differences from the original Papworth protocol, due to the possibility to perform ante-mortem interventions that are allowed in Belgium but banned by law within the UK [49]. In particular, WLST is undertaken in the OT, and donors undergo *ante-mortem* heparinization and femoral cannulation and connection to veno-arterial ECMO. Once death is declared after a 5-min no-touch period, a sternotomy is rapidly performed and the pericardium opened; the three aortic arch vessels are then clamped to exclude the cerebral circulation before full-flow NRP is established and the patient is reventilated [49]. NRP-CS has also been used in Belgium for pediatric heart transplantation from Maastricht III DCD donors [50] and in Maastricht V DCD donors after euthanasia [51]. Spanish DCD HT program started in 2020, and like the Belgian experience, WLST is always performed in the OT and, in accordance with the Spanish regulatory framework, the administration of heparin and cannulation of the femoral vessels is allowed immediately before the WLST [52,53]. In Italy, the first HT from a DCD donor was performed in 2023 using NRP-CS. Of note, many differences exist compared to other European countries: electrical asystole rather than mechanical asystole is used to ascertain cardiac death, the mandatory no touch period is 20 min, and the fWIT limit is 45 min instead of 30 min [54].

The NRP-CS technique for DCD heart recovery has also been used in the US since 2020 [55,56]. Due to regulations limiting invasive premortem hemodynamic and anatomic testing, donor inclusion in the Vanderbilt protocol was limited to donors aged < 35 years, agonal phase < 35 min and predicted cold ischemic time < 240 min. NRP was performed with a modified ECMO circuit with a blood reservoir and perfusion time was limited to 60 min to minimize the risks of ongoing bleeding and the need for blood transfusions to maintain adequate pump flows [55]. In the DCD protocol published by Smith and colleagues, after declaration of donor death, a sternotomy was performed, and the three aortic arch vessels were clamped to exclude cerebral perfusion. Standard central cannulation via the ascending aorta and right atrium was then performed, and cardiopulmonary bypass (CPB) was initiated to establish NRP [56]. A left ventricular vent was placed to decompress the heart. The donor was reintubated and ventilated. A pulmonary artery catheter and a transesophageal probe were placed to evaluate cardiac structure and function. Target goals were: CI > 3 L/min/m^2^ and mean arterial pressure (MAP) 70–90 mm Hg. If necessary, the protocol allowed for infusion of dobutamine to a maximum of 5 mg/kg/mg and of vasopressors titrated to maintain the target MAP. At 30 min intervals of NRP, CPB was weaned, and cardiac assessment was performed. The donor heart was considered acceptable for transplantation if all of the following parameters were met: MAP > 60 mm Hg, CVP < 12 mm Hg, PAPs < 40 mm Hg, PCWP < 12 mm Hg, SVO2 > 65%, CI > 2.2 L/min/m^2^, LVEF > 50% with normal right ventricular function, mitral valve lateral S wave 10 cm/s, and VTI > 15 cm. If the donor heart did not meet the aforementioned criteria, CPB was resumed for an additional 30 min, followed by reassessment of cardiac function. The protocol allowed for up to a total of 3 h on CPB to evaluate the heart for potential transplantation [56].

Previous studies from both Europe and the US found no differences in short- and long-term outcomes between cardiac allografts from TA-NRP DCD compared with DBD donors [48,57,58,59]. In the Vanderbilt experience, 385 adult recipients underwent HT between January 2020 and January 2023, including 122 (32%) from DCD donors, 83% of which were recovered with the use of TA-NRP [57]. DCD donors were younger and had fewer comorbidities than DBD donors. DCD recipients were less often hospitalized and had less temporary mechanical circulatory support (MCS) before transplantation compared with DBD recipients. There were no significant differences between groups in 1-year survival, incidence of severe PGD, treated rejection during the first year, or likelihood of cardiac allograft vasculopathy at 1 year after transplantation [57]. A propensity score matching analysis of 415 heart recipients (275 in the DBD group and 140 in the DCD-NRP group) transplanted between January 2020 and June 2024 showed no differences in severe PGD, MCS, right heart dysfunction, vasoactive inotropic scores, and 30-day, 1- and 3-year survival between groups [58].

A Spanish multicenter, nationwide, prospective study describes the short-term outcomes of adult patients receiving a DCD HT obtained via TA-NRP-CS compared to DBD donors. During 2020–2023, 98 adult DCD and 347 DBD HTs were performed across 11 centers. Thirty-day and 1-year survival were 94.9% and 88.8% in the DCD group vs. 93.7% and 87.3% in the DBD group, respectively, while severe PGD was observed in 13.3% DCD recipients vs. 15.0% DBD recipients [59].

The NRP-CS technique is cheaper and enables earlier reperfusion of all donor organs, and most importantly, it allows a functional and quality assessment of the heart with echocardiography, direct cardiac output and pressure measurements, which are essential to predict function within the recipient and reduce the risk of PGD.

Recent studies showed that preservation of the graft after NRP may benefit from the Paragonix SherpaPak Cardiac Transport System (Paragonix Technologies, Cambridge, MA, USA), which provides uniform cooling with temperatures consistently >4 °C and safe preservation of DCD grafts for ≥ 3.5 h [60].

Similarly, Williams and colleagues showed that 10 °C versus ice preservation following TA-NRP was associated with a significantly reduced risk of severe PGD, severe right ventricular failure, lower vasoactive inotropic scores at 24 h, decreased incidence of recipient renal replacement therapy, shorter intensive care unit length of stay, and reduced 6-month mortality [61].

### Ethical Issues

NRP allows reperfusion and functional assessment of the heart, thereby ensuring that only viable hearts are selected for transplantation. However, this technique continues to raise ethical concerns due to the risk of brain reperfusion via collateral circulations to the vertebrobasilar system during TA-NRP, despite the clamping of supra-aortic trunks, that could invalidate the diagnosis of death [62]. While in A-NRP, the prevention of cerebral perfusion is assured by the presence of an intra-aortic balloon [23], in TA-NRP, concerns have been raised about the possibility that clamping of the supra-aortic trunks could not be sufficient to exclude cerebral perfusion. In a series of three TA-NRP cases, where the arch vessels were clamped and the cephalad ends either vented to the atmosphere or drained to negative pressure, the authors described in one donor an estimated flow from the cephalad ends up to 50 mL/min, which is below the threshold required for the resumption of neuronal activity [62]. Severing the distal ends of the clamped brachiocephalic arteries has been proposed to facilitate blood drainage and allow intravascular pressure to equalize with atmospheric pressure [61], and this technique has also been endorsed by the nationwide Spanish protocol for TA-NRP [63]. Invasive monitoring of the intracranial blood pressure during two TA-NRP procedures using this technique showed the absence of brain perfusion even when heart function was completely resumed and TA-NRP was weaned off [64]. The use of bispectral index and transcranial Doppler ultrasound has been proposed to monitor brain perfusion. One study based on the use of intraoperative transcranial Doppler demonstrated that TA-NRP did not restore brain blood flow [65]; however, other authors suggested avoiding TA-NRP in favor of the simultaneous use of DPP for heart and lung procurement and A-NRP for abdominal organ procurement [66,67]. Conversely, a recent international consensus statement concluded that there is no evidence to support the requirement for adjunctive brain monitoring during TA-NRP [68]. TA-NRP is currently allowed in Italy, Spain, the Netherlands and some centers in the US, while it has been suspended in the UK and Belgium and is prohibited in Canada, Australia and New Zealand. Further research enrolling significantly more cases and a transatlantic collaboration have been advocated to definitively establish the absence of brain blood flow or perfusion during TA-NRP [69].

*Ante-mortem* interventions, such as heparin administration and vessel cannulation, also vary widely between countries. In the UK, the Netherlands and South Wales, ante-mortem interventions including heparin administration are strictly forbidden. In the US, *ante-mortem* heparin administration is permitted, while in Italy, *ante-mortem* heparin administration and identification of vessels to speed up post-mortem cannulation is common practice. In Spain, *ante-mortem* peripheral cannulation and heparin administration are allowed. As these procedures have no direct medical benefit to the prospective donor and carry inherent clinical risks, they are also considered ethically controversial.

## 8. Comparison Between DPP and NRP

There is growing literature comparing short- and long-term outcomes of HT from cDCD donors procured with TA-NRP or DPP. Some studies reported no difference in recipient survival between the TA-NRP and DPP techniques for heart recovery after cDCD HT. The analysis of 118 DCD HT (87 NRP, 31 DPP 31) performed from February 2020 to January 2023 at a single center showed that donors recovered using NRP were younger (25 years vs. 31 years) and had shorter travel distance compared to DPP donors. There was no difference in the incidence of severe PGD; however, LVEF at 7 days after transplantation was significantly higher in the NRP group. There was no difference in inotrope scores at 24 and 72 h or in 30-day and 1-year survival between the two groups [70]. Similarly, a propensity-score matching analysis of 318 DCD heart transplants (216 procured via DPP and 102 via NRP) performed from 2019 to 2021 found no significant difference in 1-year post-transplant survival (93.1% for NRP vs. 91.1% for DPP, *p* = 0.79) between procurement techniques [71]. An analysis of the United Network for Organ Sharing (UNOS) database demonstrated similar rates of PGD between TA-NRP and DPP, with a severe PGD rate of 9.4% and 9.7%, respectively [72]. One of the largest American series of 918 heart donors and recipients, including 622 (68%) DPP and 296 (32%) NRP transplantations, showed improved short-term survival associated with NRP. After adjustment, DCD HT with NRP was independently associated with improved survival [73]. Similarly, a recent international study of 504 cDCD HT from 22 centers in Belgium, Spain, the UK, and the US showed better outcomes with TA-NRP compared to DPP [74]. In this study, TA-NRP was associated with a 60% reduction in the incidence of severe PGD and a 40% reduction in the incidence of acute cellular rejection requiring treatment compared to DPP. These differences were observed despite similar donor and recipient demographics in both TA-NRP and DPP groups [74]. A retrospective analysis of the UNOS database evaluated the results of 11,767 DBD, 507 DPP and 265 NRP HT performed from December 2019 to September 2023 and demonstrated significantly lower rates of recipient survival in the DPP group at 3 years [75]. A recent report of 575 heart recipients of cDCD donors recovered with DPP showed a potential detrimental effect of this technique, especially for extended *ex situ* perfusion >8 h. Of note, 1- and 3-year outcomes showed worse mortality in recipients of longer-perfused hearts [76].

Concerns exist about the potential impact of heart procurement strategies on thoraco-abdominal organ utilization and outcomes. Besides offering a cheaper alternative to organ recovery than DPP, TA-NRP has been associated with better utilization of thoracic and abdominal organs from cDCD donors and with superior recipient outcomes [77]. Thomas and colleagues showed that the transplantation rate of concurrently procured cDCD livers was higher with TA-NRP compared to DPP, with no difference in 6-month liver graft failure rate [78], and recipients of kidneys procured with TA-NRP had less delayed graft dysfunction, shorter length of stay, and lower serum creatinine at discharge [78]. However, 6-month recipient survival after cDCD liver and kidney transplantation was similar in the TA-NRP and DPP groups.

Previous studies also raise concerns about the potential of TA-NRP to compromise the quality and utilization of lung allografts by inducing inflammatory-mediated lung injury, pulmonary edema and hampering lung function assessment [79,80]. A recent international study found no difference in lung utilization between TA-NRP and DPP [74]. Another report on 85 DBD and 23 cDCD TA-NRP lung transplants performed between 2022 and 2024 suggested that TA-NRP does not negatively impact lung function, as PGD grade 3 rates and overall survival were not significantly different between cDCD and DBD recipients [81]. An analysis of the UNOS database on 627 cDCD donors whose hearts were procured (211 TA-NRP, 416 DPP) between 2019 and 2022 showed no difference in lung utilization rates between the two techniques. Additionally, lung recipients from TA-NRP donors required numerically lower rates of ECMO and mechanical ventilation at 72 h, and 6-month survival was similar between groups [82]. Other reports demonstrated that outcomes from lungs recovered using TA-NRP are similar to those recovered using DPP [83,84], and a Spanish study reported significantly lower rates of PGD in lungs recovered using TA-NRP compared to those recovered during A-NRP [85].

To date, the comparison between TA-NRP and DP-HOPE has been reported only by a single center. In this study, cDCD Maastricht category III and V donors were considered for heart transplant, 8 with DP-HOPE and 10 with TA-NRP-CS, showing 100% survival in both groups [86].

The relative advantages and disadvantages of each approach are summarized in Table 2. Differences in the reported clinical outcomes need careful interpretation. Clinical evidence derives from feasibility reports and case series, single-center observational studies, registry analyses and propensity-matched studies, while no randomized data are available. Many studies involve younger donors, short ischemic times, experienced centers, and highly selected recipients, which may limit generalizability. Apparent advantages of one technique over another may reflect donor age and selection criteria, recipient acuity and risk, ischemic time, center volume and experience, *ex situ* perfusion duration, different preservation pathways after recovery, shorter transport distance, PGD definitions and organ utilization bias.

## 9. Rapid Recovery with Extended Ultraoxygenated Preservation

Given the high cost and complexity of current cDCD heart recovery strategies, as well as ethical concerns surrounding TA-NRP, a new technique for the recovery of hearts from cDCD donors that obviates the need for TA-NRP or *ex situ* perfusion systems has been proposed, called Rapid Recovery with Extended Ultraoxygenated Preservation (REUP) [87]. Briefly, after the declaration of death, the sternotomy is performed, the aorta is cross-clamped and a flush circuit is established to perform a controlled, extended, ultraoxygenated flush of the donor heart. The flush circuit consists of an extracorporeal circuit with an additional reservoir and a heater and cooler. The extracorporeal circuit is primed with 2 units of crossed packed red cells, 500 cc of PlasmaLyte crystalloid solution, 2 L of del Nido cardioplegia solution, 30,000 UI of heparin, mannitol, albumin, solumedrol, antibiotics, levothyroxine, and other additives. The preservation solution is oxygenated through the extracorporeal circuit and sweep gas at a rate of 2 L per minute, and 100% fraction of inspired oxygen is maintained for 5 min. The heater and cooler ensured a flush temperature of 4 °C. The solution is administered at a mean aortic root pressure of 80 mm Hg over a period of approximately 10 to 12 min [87]. In the first three reported cases in which this method was used, the hearts were transplanted successfully with normal biventricular function, no evidence of acute cellular or antibody-mediated rejection, and excellent early postoperative outcomes. No adverse events were reported during the perioperative period [87]. A case series of 24 patients who underwent REUP-recovered DCD adult heart transplant at a single high-volume heart transplant center in the United States from November 2024 to July 2025 has been recently reported [84]. The mean donor age was 32 years, the mean time from initial declaration of donor death to flush was 9 min and 15 donor hearts (60%) had a total ischemic time longer than 4 h. Among recipients, 30-day survival was 96%. Only one patient (4%) had severe PGD, and one other patient (4%) had secondary graft dysfunction [88]. This emerging strategy is promising; however, it requires multicenter validation.

## 10. Beating Heart Transplantation

Donor hearts are procured following cold cardioplegic arrest and are subsequently transported and then implanted under hypothermic and hypoxic conditions. Thus, graft ischemia and reperfusion injury (IRI) are considered an unavoidable part of organ transplantation. Importantly, IRI contributes to PGD, which is a leading cause of early morbidity and mortality following HT [89]. A previous report showed the safety and feasibility of ischemia-free beating heart transplantation, with a DBD donor heart procured, preserved, and implanted under a continuously perfused, normothermic, oxygenated, beating state [89,90]. DCD HT usually requires two cardioplegic arrests, compared with DBD, which involves only one cardioplegic arrest, thus involving two periods of warm ischemia and two periods of cold ischemia, exposing the graft to multiple rounds of IRI, potentially increasing the risk of PGD after DCD HT. Krishnan and colleagues developed a method of performing DCD HT with uninterrupted coronary perfusion after heart procurement, keeping the heart beating through implantation [91]. The heart was procured with the DPP technique as described above. Once the donor organ arrived at the recipient OT, a modified setup that allows for tandem OCS and CPB was initiated. Antegrade perfusion with warm blood, now coming from the CPB circuit via a cardioplegia cannula pressurized at 300 mmHg for an estimated root pressure of 100 mmHg, was initiated in the donor heart, and a cross-clamp was placed just superior to the antegrade cannula to ensure direction of flow into the coronary arteries. The heart was then separated from the OCS device and transferred sterilely to the operating table, while perfused by the CPB circuit with recipient warm blood, paced and beating. Once the left atrial cuff and aortic anastomosis were completed, both the donor organ and recipient aortic cross-clamps were removed, with perfusion now being provided by the aortic cannula. The results of the first 10 recipients undergoing DCD HT with the beating heart implantation method showed 100% survival and no PGD or need for temporary MCS or re-transplantation [92]. This novel strategy, avoiding additional ischemic time after procurement and allowing the heart to be implanted while beating and perfused with warm blood, appears safe and may reduce the IRI and the rates of PGD; however, the early published experience needs further validation.

## 11. Future Perspectives

The use of extended criteria donors and cDCD donors has demonstrated their potential to safely expand the pool of donor hearts available for HT. However, organ acquisition costs vary for DBD and DCD HT, as DCD shows both higher baseline acquisition costs and greater financial volatility compared to DBD. On average, DCD HT costs 57% more than DBD HT. Of note, perfusion and preservation technologies are 43% higher, transportation is 64% higher and declined cases are 31% higher for DCD compared to DBD [93]. Artificial intelligence may assist in increasing the availability of heart grafts by optimizing donor-recipient matching and predicting patient outcomes given various donor–recipient characteristics, thus reducing organ discards and resource waste. The use of ML models has been demonstrated to be accurate in predicting mortality and survival after HT, waitlist mortality and the risk of allograft rejection [94]. Concerns still remain regarding the quality of hearts procured in cDCD donors, due to injury provoked by warm ischemia and subsequent reperfusion. *Ex situ* normothermic perfusion can reduce ischemic injury compared to CS and allows for assessment of organs during transport and before implantation via sampling of perfusion solution, visual inspection, and coronary angiography. Emerging studies highlighted the potential of *ex situ* machine perfusion for drug delivery and allograft “reconditioning” during transport to optimize post-transplant outcomes [95]. Genomic and metabolomic profiling showed the potential to identify new biomarkers of ischemia-reperfusion injury that could predict allograft outcomes and play a potential role for future therapeutic interventions [96].

## 12. Conclusions

**Figure 4:**
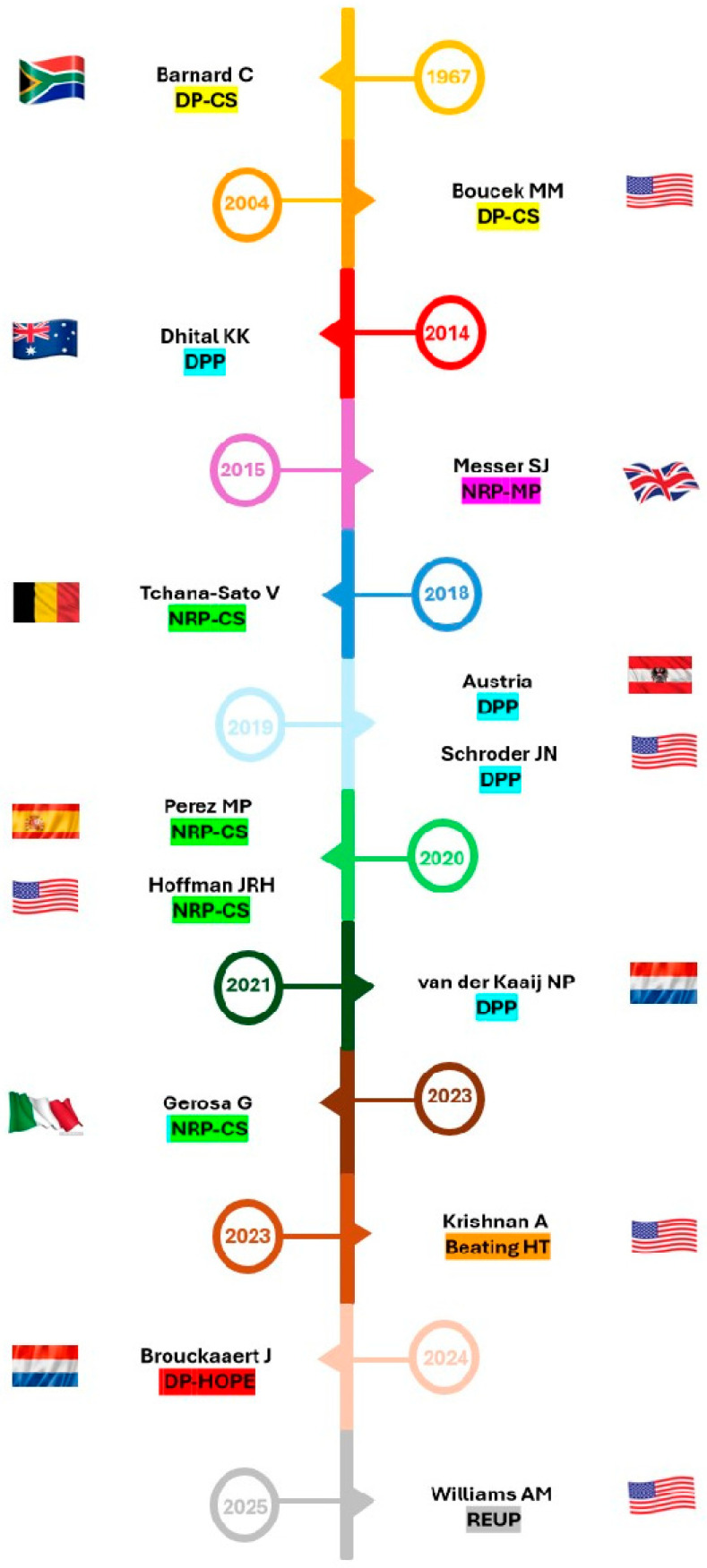
Timeline of worldwide cDCD heart transplantation. CS: cold storage; DP: direct procurement; DPP: direct procurement and perfusion; HOPE: hypothermic oxygenated perfusion; NRP: normothermic regional perfusion; MP: machine perfusion; REUP: Rapid Recovery with Extended Ultraoxygenated Preservation. [17,24,25,32,33,34,44,48,51,53,54,83,87].

Available strategies for DCD HT face financial, logistical and ethical challenges, translating into a heterogeneous landscape of cDCD protocols across European and non-European countries (Figure 4). Current clinical evidence is based on retrospective registry data and single-center observational studies and, to date, no published RCT has directly compared short- and long-term outcomes of HT from cDCD donors recovered with DPP or TA-NRP. New strategies are emerging to reduce heart injury provoked by warm ischemic time; however, further studies are needed to validate their safety and effectiveness. In conclusion, cDCD HT has evolved from an experimental approach to an established clinical practice in experienced centers. Continuous technological advances are likely to further expand donor utilization and improve recipients’ clinical outcomes. However, future multicenter prospective studies and randomized trials are needed to determine the optimal reperfusion strategy.

## Figures and Tables

**Table 2:** Summary of the characteristics, advantages and disadvantages of each reperfusion technique.

	DPP	TA-NRP	DP-HOPE
**Technique**	*Ex situ*	*In situ*	*Ex situ*
**Preservation method**	Normothermic machine perfusion	ECMO/CPB	Hypothermic machine perfusion
**Temperature**	34 °C	37 °C	8–10° C
**Functional assessment**	Yes	Yes	No
**Ischemic time**	fWIT < 30 min	fWIT < 45 min	fWIT < 30 min
**Transport method**	Normothermic machine perfusion	Cold storage or normothermic machine perfusion	Hypothermic machine perfusion
**Current clinical evidence**	Single-center observational studies Registry analyses, Propensity-matched studies	Single-center observational studies Registry analyses, Propensity-matched studies	Feasibility reports and case series, Single-center observational studies
**Survival outcomes**	Excellent	Excellent	Favorable early results
**Geographical use**	Belgium, UK, US, Canada, Australia	Spain, Italy, Netherlands, US	Belgium
**Commercial availability**	Yes	Yes	No
**Costs**	High	Moderate	High
**Logistical complexity**	High	Moderate/high	Moderate
**Ethical issues**	None	Risk of brain reperfusion	None
**Advantages**	No ethical concern, long distance transport	Functional assessment, highly effective for multi-organ recovery	No ethical concern, long distance transport
**Disadvantages**	High costs and complex logistics	Ethical concerns	No functional assessment

CPB: cardiopulmonary bypass; DP-HOPE: direct procurement and hypothermic oxygenated perfusion; DPP: direct procurement and perfusion; ECMO: extra-corporeal membrane oxygenation; fWIT: functional warm ischemic time; TA-NRP: thoraco-abdominal normothermic regional perfusion.

## Data Availability

No new data were created or analyzed in this study. Data sharing is not applicable to this article.

## Abbreviations

The following abbreviations are used in this manuscript:

A-NRP	Abdominal Normothermic Regional Perfusion
CAV	Cardiac Allograft Vasculopathy
cDCD	Controlled Donation after Circulatory death
CI	Cardiac Index
CPB	Cardiopulmonary Bypass
CS	Cold Storage
CVP	Central Venous Pressure
DBD	Donation After Brain Death
DCD	Donation After Circulatory death
DP	Direct Procurement
DPP	Direct Procurement and Perfusion
ECG	Electrocardiogram
ECMO	Extra-Corporeal Membrane Oxygenation
fWIT	Functional Warm Ischemic Time
HOPE	Hypothermic Oxygenated Perfusion
HT	Heart Transplantation
IRI	Ischemia Reperfusion Injury
ISHLT	International society of Heart and Lung Transplantation
LVEF	Left Ventricular Ejection Fraction
MAP	Mean Arterial Pressure
MCS	Mechanical Circulatory Support
NMP	Normothermic Machine perfusion
NRP	Normothermic Regional Perfusion
OCS	Organ Care System
OT	Operating Theatre
PAPs	Pulmonary Artery Pressure Systolic
PCWP	Pulmonary Capillary Wedge Pressure
PGD	Primary Graft Dysfunction
RCT	Randomized Clinical Trial
REUP	Rapid Recovery with Extended Ultraoxygenated Preservation
SVO2	Venous Oxygen Saturation
TA-NRP	Thoraco-Abdominal Normothermic Regional Perfusion
tWIT	Total Warm Ischemic Time
UK	United Kingdom
US	United States
VTI	Velocity Time Integral
WLST	Withdrawal of Life Sustaining Therapy
